# Alternative antibiotics for the treatment of bacterial meningitis in children: a systematic review of efficacy and safety

**DOI:** 10.1016/j.jped.2025.05.002

**Published:** 2025-06-12

**Authors:** Ana Clara Valente de Alencar, Athus Graziani Apollaro Rego, Carolina Soares Chady, Alisson Gabriel Maués Miranda, Lara Fernanda Alves de Souza, João Vitor Martins Pinto, Bárbara de Souza Maia do Nascimento, Camili Giseli Oliveira de Menezes, Isa Mendes Moreira, José Carlos Favacho Furlan, Ester Carolina Vieira Maia, Maria Fernanda Nakayama Martins, Nayara Cristina Cardoso da Silva, Paula do Socorro de Oliveira da Costa Laurindo, Luis Edilson de Azevedo Ferreira, Antônio Rafael Quadros Gomes, Heliton Patrick Cordovil Brígido

**Affiliations:** a Centro Universitário Metropolitano da Amazônia (UNIFAMAZ), Curso de Medicina, Belém, PA, Brazil; b Universidade Federal do Pará (UFPA), Programa de Pós-Graduação em Ciências Farmacêuticas (PPGCF), Belém, PA, Brazil; c Universidade do Estado do Pará (UEPA), Centro de Ciências Biológicas e da Saúde (CCBS), Tucuruí, PA, Brazil; d Universidade Federal do Pará (UFPA), Programa de Pós-Graduação em Biodiversidade e Biotecnologia (BIONORTE), Belém, PA, Brazil; e Universidade Federal do Pará (UFPA), Conselho Nacional de Desenvolvimento Científico e Tecnológico (CNPq), Belém, PA, Brazil

**Keywords:** Bacterial meningitis, Antibiotic safety, Ceftriaxone, Antibiotics, Clinical efficacy

## Abstract

**Objective:**

This systematic review aimed to evaluate the efficacy and safety of alternative antibiotics and different standard treatment regimens for bacterial meningitis in children, considering the increasing antimicrobial resistance and the need for adapted therapeutic options. To justify the use of alternative antibiotics, the authors analyzed the specific efficacy of ampicillin, chloramphenicol, cefuroxime and meropenem, which showed potential to overcome cases of antimicrobial resistance.

**Data sources:**

A search was performed in databases such as PubMed, Scopus, Web of Science and Cochrane Library, without data restrictions, including planned clinical trials that compared alternative antibiotics and different standard treatment regimens, such as ceftriaxone, in children with bacterial meningitis. Inclusion criteria include studies reporting cure rates, complications and safety of treatments.

**Summary of results:**

An analysis of 14 studies, totaling 2,014 children, indicated that antibiotics such as ampicillin, chloramphenicol, cefuroxime and meropenem had comparable efficacy and safety to standard treatment regimens. The review showed that, in many cases, alternative regimens and shorter treatment durations could be effective, without significantly increasing complications or mortality.

**Conclusion:**

The results suggest that alternatives to standard treatment, such as ampicillin, chloramphenicol, cefuroxime and meropenem, are viable and safe options for the treatment of bacterial meningitis in children. These results help to adapt clinical practices, especially in settings with high antimicrobial resistance and resource limitations, by providing evidence for shorter and equally effective treatment regimens.

## Introduction

Bacterial meningitis is one of the most serious infections affecting the central nervous system, with significant impacts on global child health. It is estimated that in 2022, there were around 2.5 million cases of meningitis worldwide, resulting in approximately 250,000 deaths, many of which were in children under five years of age.^1^ This condition can be caused by several bacteria, the most common being *Streptococcus pneumoniae, Neisseria meningitidis* and *Haemophilus influenzae* type b, with variations depending on the region and the vaccination status of the population.^1^

In response to the global burden of meningitis, the World Health Organization (WHO) launched the initiative “Defeating Meningitis by 2030 – Global Roadmap”, which outlines strategic actions to eliminate meningitis epidemics, reduce mortality, and improve the quality of life for survivors. This roadmap emphasizes not only vaccination but also timely diagnosis, effective treatment, and strong surveillance systems, especially in vulnerable pediatric populations. The present review aligns with the roadmap's objectives by contributing evidence to support therapeutic decision-making and highlight safe and effective antibiotic alternatives in resource-limited settings.^1^

In Brazil, for example, cases of bacterial meningitis are still a major cause of child mortality, despite vaccination campaigns. According to recent data from the Ministry of Health, the country recorded more than 15,000 cases of meningitis in 2021, with bacterial meningitis accounting for around 60% of these cases.^2^

The standard treatment for bacterial meningitis involves the immediate administration of broad-spectrum antibiotics, such as ceftriaxone and cefotaxime, in addition to intensive supportive measures. However, with the increase in antimicrobial resistance and the variability of clinical outcomes, there is a growing interest in the evaluation of alternative antibiotics and combination regimens that may offer greater efficacy or reduce adverse effects.^3^ New classes of antibiotics and adjuvant therapies, such as beta-lactamase inhibitors and carbapenems, have been investigated with promising results in recent studies.^4^

Given the increase in antimicrobial resistance worldwide, it is essential to search for alternatives that guarantee the efficacy of treatment and reduce the side effects associated with first-line antibiotics.^5^^,^^6^ Recent studies have suggested that alternative antibiotics, such as meropenem and chloramphenicol, may play an important role as substitutes or complements to standard treatments, particularly in settings with limited resources or high bacterial resistance.^1^^,^^3^

Systematic reviews comparing the efficacy and safety of alternative antibiotics in the treatment of bacterial meningitis are scarce. Considering the long-term impact that this disease can have, particularly on a child neurological development, it is essential that new therapies are rigorously evaluated.^5^ This study aims to fill this gap by conducting a systematic review of the literature on the use of alternative antibiotics in children with bacterial meningitis. The aim is to provide a solid evidence base that contributes to clinical practice, assisting in the selection of more effective and safe treatments for this vulnerable population.

## Methodology

### Literature source and search strategy

This study was conducted as a systematic review registered in PROSPERO (ID: CRD42024528772) and structured according to the PRISMA (Preferred Reporting Items for Systematic Reviews and Meta-Analyses) guidelines to ensure rigor and transparency. The objective was to comparatively evaluate the efficacy and safety of alternative antibiotics in the treatment of childhood bacterial meningitis. The research question was formulated using the PICO model, establishing the following focus: *“In children up to 12 years of age with bacterial meningitis, which alternative antibiotics demonstrate comparable or superior efficacy and safety to first-line treatment in terms of cure rate, mortality, complications and adverse events?”.*

The literature search was conducted in four major databases: PubMed, Scopus, Web of Science, and the Cochrane Library, using specific MeSH terms such as *bacterial meningitis, antibiotic therapy, children*, and *randomized clinical trials*. The search was restricted to studies published in English and was supplemented by a manual search of the reference lists of included articles. Randomized clinical trials comparing alternative antibiotics with standard treatment regimens — including variations in dosage and duration of ceftriaxone — were considered eligible, particularly those reporting key clinical outcomes such as therapeutic success, mortality, adverse events, and complications.

Search strategies were developed using controlled vocabulary (MeSH terms) and Boolean operators, tailored to the syntax of each database. Combinations of terms included: *“bacterial meningitis” and “antibiotic therapy” and “children”, “bacterial meningitis” and “randomized controlled trial” and “treatment efficacy”*, and *“ceftriaxone” or “chloramphenicol” or “cefuroxime” or “meropenem” and “meningitis” and “pediatrics”*. Filters were applied based on language, study type, and population characteristics. The complete list of search strategies used in each database is provided in Appendix A.

### Inclusion and exclusion criteria

This systematic review included randomized clinical trials that evaluated children aged up to 12 years diagnosed with bacterial meningitis. Eligible studies encompassed neonates (< 28 days), infants (1–12 months), toddlers, and older children, provided that age-specific data were clearly reported. However, as not all studies stratified outcomes by age subgroup, age-based subgroup analysis (e.g., neonates vs. older children) was only partially feasible and is further discussed in the limitations section.

Trials were included if they compared alternative antibiotics with standard-of-care agents, such as ceftriaxone or cefotaxime, and addressed different administration regimens of ceftriaxone (e.g., variations in dosage and treatment duration). In addition, studies had to report at least one of the following clinical outcomes: treatment efficacy (e.g., cure rates) or safety data (e.g., mortality, adverse events, and complications). Only studies with full-text access available were considered.

Studies were excluded if they: (1) included adult populations; (2) did not clearly report outcomes related to the research objectives; or (3) were non-randomized designs, including reviews, case reports, or observational studies that did not meet the inclusion criteria.

### Data selection and extraction

Study selection was conducted in two phases. In the first phase, titles and abstracts were screened independently by two reviewers. In the second phase, the full texts of the selected articles were evaluated to verify whether they met the predefined inclusion criteria. The selection process was carried out independently by both reviewers. Discrepancies were initially resolved through discussion, and when consensus could not be reached, a third reviewer was consulted for a final decision. Inter-rater agreement was assessed using Cohen's kappa coefficient, which yielded a value of 0.81, indicating excellent concordance.

Data extracted from each study included the following characteristics: author, year of publication, study design, and number of participants. Additional data were collected on the study population (such as age, diagnosis, and comorbidities), the interventions (antibiotics used), and the clinical outcomes evaluated, including clinical cure, mortality, complications, and adverse events.

The primary outcome was defined as the clinical cure rate at the end of treatment. Secondary outcomes included the incidence of complications, the main pathogens involved, and the occurrence of adverse reactions to the antibiotics under investigation. Clinical cure was defined as the reduction or disappearance of symptoms, primarily assessed at the end of treatment or during post-treatment follow-up.

The methodological quality of the included studies was independently assessed by two reviewers using the Cochrane Risk of Bias tool for Randomized Controlled Trials (RoB 2). The domains evaluated included: (i) bias arising from the randomization process, (ii) bias due to deviations from intended interventions, (iii) bias due to missing outcome data, (iv) bias in the measurement of the outcome, (v) bias in the selection of the reported result, and (vi) bias related to blinding of participants and personnel. Disagreements were resolved by consensus. The results of this assessment are summarized in Table 1.

**Table 1:** Risk of bias assessment of included studies.

Study	Random sequence generation	Allocation concealment	Blinding of participants and personnel	Blinding of outcome assessment	Incomplete outcome data	Selective reporting	Overall risk of bias
Del Rio et al.^7^	Low	Unclear	Low	Low	Low	Low	Low
Aronoff et al.^8^	Low	Low	Unclear	Low	Low	Low	Low
Barson et al.^9^	Unclear	Unclear	Unclear	Unclear	Low	Low	Moderate
Girgis et al.^10^	Low	Unclear	Unclear	Low	Low	Low	Moderate
Kumar & Verma^11^	Low	Low	Low	Low	Low	Low	Low
Sharma et al.^12^	Unclear	Unclear	Unclear	Unclear	Low	Unclear	Moderate
Pécoul et al.^13^	Low	Low	Low	Low	Low	Low	Low
Marks et al.^14^	Low	Low	Unclear	Low	Low	Low	Low
Schaad et al.^15^	Low	Low	Low	Low	Low	Low	Low
Klugman et al.^16^	Low	Low	Low	Low	Low	Low	Low
Peltola et al.^17^	Unclear	Unclear	Unclear	Unclear	Low	Unclear	Moderate
Sáez-Llorens et al.^18^	Low	Low	Low	Low	Low	Low	Low
Molyneux et al.^19^	Low	Low	Low	Low	Low	Low	Low
Vaswani et al.^20^	Low	Low	Low	Low	Low	Low	Low

## Results

**Figure 1:**
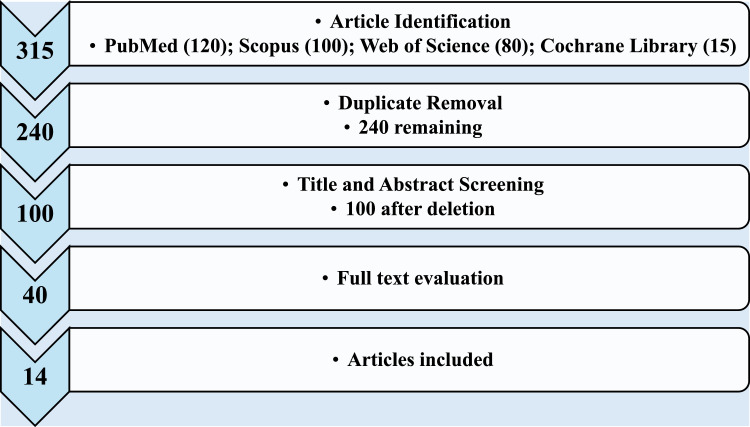
Processing flow for article selection in systematic review.

After searching the PubMed, Cochrane Library, SCOPUS and Web of Science databases, 315 articles related to the treatment of bacterial meningitis in children were identified, of which 120 were available in PubMed, 15 in the Cochrane Library, 100 in SCOPUS and 80 in Web of Science. After removing duplicate articles, 240 unique studies remained for screening. In the first phase, the analysis of titles and abstracts resulted in the exclusion of 140 articles that did not meet the eligibility criteria, such as focusing on adult populations or non-antibiotic treatments, leaving 100 articles (Figure 1).

In the second phase, 40 articles were selected for full reading. During the evaluation of the full text, 26 studies were excluded due to a lack of direct comparisons between alternative and first-line antibiotics, or due to the lack of sufficient data on relevant clinical outcomes. In the end, 14 articles fully met the inclusion criteria and were incorporated into the systematic review (Figure 1).

The selected articles cover a total of 2,014 children diagnosed with bacterial meningitis. The alternative antibiotics investigated throughout the studies included ceftriaxone, ampicillin, chloramphenicol, cefuroxime, cefotaxime, meropenem and penicillin G. Ceftriaxone was extensively evaluated and often compared with combinations of other antibiotics, such as ampicillin and chloramphenicol, in addition to different treatment durations. Among the main pathogens isolated in the studies, *S. pneumoniae, N. meningitidis* and *H. influenzae* stood out, recognized as the most common etiological agents of bacterial meningitis in children. These studies provide a solid basis for evaluating the efficacy and safety of alternative antibiotics, demonstrating that most of the regimens studied were effective in terms of both clinical cure and bacterial sterilization in the cerebrospinal fluid.

The results of the studies included in this review were organized in detail and presented in groups, based on the main antibiotics and specific therapeutic regimens, as described below.

One group of studies investigated the combination of ampicillin with chloramphenicol in comparison with the use of ceftriaxone, evaluating their efficacy and safety as alternatives in the treatment of bacterial meningitis.

In the study by Del Rio et al.,^7^ a randomized clinical trial involving 78 children diagnosed with bacterial meningitis, patients were treated with ceftriaxone (75 mg/kg followed by 50 mg/kg every 12 hours) or with a combination of ampicillin (200 mg/kg/day) and chloramphenicol (100 mg/kg/day), administered every 6 hours. After 12 hours of treatment, cerebrospinal fluid (CSF) cultures were negative in 57% of patients in the ceftriaxone group and in 42% in the ampicillin + chloramphenicol group. Clinical efficacy was 85-90% for ceftriaxone and 80-85% for the ampicillin + chloramphenicol group, with no significant differences in neurological complications between the groups (Table 2).

**Table 2:** Comparison of clinical efficacy and adverse reactions of alternative antibiotics in the treatment of bacterial meningitis in children.

Author	Number of patients treated	Drug and dose	Treatment time	Pathogens identified	Clinical efficacy (%)	Adverse reactions of each drug	Summary of main results
Del Rio et al.,^7^	Ceftriaxone: (39) Ampicilin + Chloramphenicol: 39	Ceftriaxone: 75 mg/kg Ampicilin: 200 mg/kg/day + Chloramphenicol: 100 mg/kg/day.	7 a 10 days	*H. influenzae* tipo b *S. pneumoniae* *N. meningitidis* Others	Ceftriaxone: 85-90% Ampicilin + Chloramphenicol: 80-85%	Ceftriaxone: Mild diarrhea (16 patients) Ampicilin + Chloramphenicol: Mild diarrhea (8 patients), no other ADRs.	Ceftriaxone was comparable to ampicillin + chloramphenicol in terms of clinical efficacy and safety, with greater bactericidal activity in CSF and faster bacterial sterilization.
Aronoff et al.,^8^	Ceftriaxone: (11) Ampicilin + Chloramphenicol: (8)	Ceftriaxone: 50 mg/kg Ampicilin: 200–300 mg/kg/day Chloramphenicol: 100 mg/kg/day.	10 a 14 days	*H. influenzae* tipo b *N. meningitidis* *S. pneumoniae* *E. coli* *P. aeruginosa*	Ceftriaxone: 90%. Ampicilin + Chloramphenicol: 85%	Ceftriaxone: No ADR. Ampicilin + Chloramphenicol: No serious ADRs reported.	Ceftriaxone was as effective as the combination of ampicillin and chloramphenicol in treating childhood bacterial meningitis.
Barson et al.,^9^	Ceftriaxone: (23) Ampicilin + Chloramphenicol: (22)	Ceftriaxone: 75 mg/kg Ampicilin + Chloramphenicol: 50 mg/kg e 25 mg/kg	Not specified	*H. influenzae* tipo b *S. pneumoniae* *N. meningitidis*	Ceftriaxone: 96%. Ampicilin + Chloramphenicol: 95%.	Ceftriaxone: Neutropenia, eosinophilia, thrombocytosis, diarrhea, hyperbilirubinemia. Ampicilin + Chloramphenicol: Neutropenia, eosinophilia, thrombocytosis, diarrhea and rash.	Both treatments were effective in reducing CSF bacterial load and clinical recovery, with similar reduction in bacterial counts.
Marks et al.^14^	Cefuroxime: (50) Ampicilin + Chloramphenicol: (57)	Cefuroxime: 225 mg/kg/day Ampicilin: 300-400 mg/kg/day Chloramphenicol: 75–100 mg/kg/day	7 ou 10 days	*H. influenzae* tipo b *S. pneumoniae* *N. meningitidis*	Cefuroxime: 94% Ampicilin + Chloramphenicol: 93%	Cefuroxime: Diarrhea, eosinophilia, thrombocytosis Ampicilin + Chloramphenicol: Diarrhea, rash, eosinophilia, thrombocytosis	The study concluded that both regimens are effective and safe, with cefuroxime offering the advantage of less frequent dosing.
Girgis et al.^10^	Ceftriaxone: (35) Ampicilin + Chloramphenicol: (35)	Ceftriaxone: 100 mg/kg/day Ampicilin: 160 mg/kg/day Chloramphenicol: 100 mg/kg/day	6 days	*N. meningitidis* *S. pneumoniae* *H. influenzae* tipo b *E. coli*	Ceftriaxone: 83% Ampicilin + Chloramphenicol: 74%	Ceftriaxone: Mild, self-limiting diarrhea in 4 children. Ampicilin + Chloramphenicol: No ADRs reported.	Both demonstrated comparable clinical efficacy in the treatment of childhood bacterial meningitis, with IM ceftriaxone being as effective as the standard treatment of ampicillin and chloramphenicol.
Peltola et al.^17^	Chloramphenicol: (50) Ampicilin: (50) Cefotaxime: (50) Ceftriaxone: (50)	Chloramphenicol: 100 mg/kg/day Ampicilin: 250 mg/kg/day Cefotaxime: 150 mg/kg/day Ceftriaxone: 100 mg/kg.	7 days	*Haemophilus influenzae* tipo b Meningococos Pneumococos Others	Ceftriaxone e Cefotaxime: 80-85% Ampicilin e Chloramphenicol: 75-80%	Ceftriaxone: Mild to moderate diarrhea in 19 cases. Ampicilin: Rash in 4 cases. Chloramphenicol: No ADRs. Cefotaxime: No ADRs reported.	Ceftriaxone and cefotaxime showed greater efficacy and faster sterilization of CSF than chloramphenicol and ampicillin. Chloramphenicol was less effective, and ampicillin faced problems of bacterial resistance.
Schaad et al.^15^	Ceftriaxone: (87) Cefuroxime: (84)	Ceftriaxone: 100 mg/kg/day Cefuroxime: 200 mg/kg/day.	7 a 10 days	*H. influenzae* tipo b *N. meningitidis* *S. pneumoniae*	Ceftriaxone: 94% Cefuroxime: 92%	Ceftriaxone: Diarrhea, skin rash, transient increase in liver enzymes. Cefuroxime: Diarrhea, skin rash, neutropenia.	Both ceftriaxone and cefuroxime are effective and safe in the treatment of bacterial meningitis in children, with ceftriaxone being given daily, which may be preferable in clinical settings.
Pécoul et al.^13^	Chloramphenicol: (254) Ampicilin: (274)	Chloramphenicol: 100 mg/kg Ampicilin: 200 mg/kg/day.	8 days ampicilin e 48 h chloramphenicol	*N. meningitidis* *H. influenzae* tipo b *S. pneumoniae*	Chloramphenicol: 72%. Ampicilin: 75,5%.	No serious ADRs were observed in either group.	Both showed similar efficacy, but chloramphenicol offered cost advantages and ease of administration under field conditions.
Kumar & Verma^11^	Chloramphenicol: (33) Chloramphenicol + Penicilin: (33)	Chloramphenicol: 100 mg/kg/day, Chloramphenicol + Penicilin: 100 mg/kg/day e 300.000–400.000 UI/kg/day	10 a 14 days	*N. meningitidis* *S. pneumoniae* *H. influenzae*	Chloramphenicol: 91% Chloramphenicol + Penicilin: 88%	Chloramphenicol: Thrombophlebitis (3.3% of patients) Chloramphenicol + Penicilin: thrombophlebitis (58.6% of patients).	Chloramphenicol administered alone has been found to be an effective and more convenient alternative, with a lower risk of complications. It is less expensive and easy to administer.
Klugman et al.^16^	Meropenem: (98) Cefotaxime: (92)	Meropenem: 40 mg/kg Cefotaxime: 75–100 mg/kg	7 a 14 days	*H. influenzae* *N. meningitidis* *S. pneumoniae*	Both 100% in CSF.	Meropenem: 6 patients with seizures. Diarrhea and fever. Cefotaxime: 3 patients with seizures, diarrhea and oral candidiasis.	Both antibiotics are effective in treating bacterial meningitis. Meropenem was a safe alternative, but there was an increased incidence of seizures among patients with a neurological history.
Sáez-Llorens et al.^18^	Cefepime: (43) Cefotaxime: (47)	Cefepime: 50 mg/kg Cefotaxime: 50 mg/kg	7 a 10 days	*H. influenzae* tipo b *N. meningitidis* *S. pneumoniae*	Cefepime: 95,3%. Cefotaxime: 91,5%.	Cefepime: Diarrhea in 5 patients and rash in 2 patients. Cefotaxime: Diarrhea in 9 patients and rash in 1 patient.	Cefepime was as effective and safe as cefotaxime for the treatment of bacterial meningitis in children. Both antibiotics were safe, with low rates of ADRs, neurological and auditory sequelae.
Sharma et al.^12^	Chloramphenicol + Penicilin: (12) Ceftriaxone: (11)	Chloramphenicol: 100 mg/kg/day Penicilin: 200.000 UI/kg/day Ceftriaxone: 50 mg/kg/day.	7 a 14 days	*H. influenzae* *N. meningitidis* *S. pneumoniae*	Ceftriaxone: 100%. Chloramphenicol + Penicilin: 100%.	Ceftriaxone: 2 patients with mild increase in CSF cell count on seventh day. Chloramphenicol + Penicilin: No serious ADRs observed.	The study showed that ceftriaxone IM once daily for 7 days was as effective as the regimen of chloramphenicol + IV penicillin for 14 days.
Molyneux et al.^19^	Benzylpenicillin + Gentamicin: (161) Ceftriaxone: (170)	Benzylpenicillin: 50.000 a 100.000 UI/kg Gentamicin: 6 mg/kg/day + Ceftriaxone: 50–100 mg/kg/day	5 a 14 days	*S. pneumoniae* *E. coli* *K. pneumoniae* *A. baumanni .*	Benzylpenicillin + Gentamicin: 83,5% Ceftriaxone: 83,5%	Ceftriaxone: 14% of patients with jaundice. Benzylpenicillin + Gentamicin: 6.5% of patients with jaundice.	Both ceftriaxone and benzylpenicillin + gentamicin are safe and effective in treating serious bacterial infections in newborns.
Vaswani et al.^20^	Ceftriaxone: (48) Vancomycin: (48)	Ceftriaxone: 100 mg/kg/day Vancomycin: 60 mg/kg/day	7 ou 10 days	*S. pneumoniae* *N. meningitidis* *H. influenzae*	Ceftriaxone: 85,42%. Vancomycin: 87,5%.	4 cases of nosocomial sepsis were reported (2 in each group), with no significant difference between groups.	The shorter 7-day treatment was as effective as the 10-day course for children with pyogenic meningitis, making it a viable and safe option.

ADR, adverse drug reaction; IM, Intramuscular; IV, Intravenous; h, hours; CSF, Cerebrospinal fluid.

In the study by Aronoff et al.,^8^ which included 19 children over 2 months of age, patients were treated with ceftriaxone (50 mg/kg every 12 hours) or with the combination of ampicillin (200-300 mg/kg/day) and chloramphenicol (100 mg/kg/day). The bacteriological cure rate was 90% in the ceftriaxone group and 85% in the ampicillin + chloramphenicol group. Ceftriaxone provided a faster recovery, with a lower incidence of neurological sequelae. Both therapeutic regimens demonstrated high clinical efficacy, with no reported deaths (Table 2).

The clinical trial by Barson et al.^9^ involved 45 children aged 3 months to 5 years who were treated with ceftriaxone (75 mg/kg followed by 50 mg/kg every 12 hours) or with the combination of ampicillin (50 mg/kg) and chloramphenicol (25 mg/kg), administered every 6 hours. After 18 hours of treatment, 65% of patients treated with ceftriaxone and 58% of those treated with the ampicillin + chloramphenicol combination had sterile CSF cultures. Clinical cure was observed in 96% of patients in the ceftriaxone group and in 95% of the ampicillin + chloramphenicol group, with no significant differences in symptom resolution or complications (Table 2).

The clinical trial by Girgis et al.^10^ was conducted in Egypt with 70 children aged 4 months to 12 years, comparing ceftriaxone (100 mg/kg/day) administered intramuscularly with the combination of ampicillin (160 mg/kg/day) and chloramphenicol (100 mg/kg/day). Complete recovery without sequelae was observed in 83% of patients treated with ceftriaxone and in 74% of those treated with ampicillin + chloramphenicol. Mortality was slightly lower in the ceftriaxone group (6 deaths) compared to the ampicillin + chloramphenicol group (9 deaths) (Table 2).

Kumar and Verma^11^ investigated 70 children in a hospital in a developing country, comparing chloramphenicol alone (100 mg/kg/day) with the combination of chloramphenicol and penicillin (300,000–400,000 IU/kg/day). Clinical efficacy was similar in both groups, with a 91% cure rate in the chloramphenicol alone group. However, the group treated with the combination had a higher incidence of thrombophlebitis (Table 2).

Sharma et al.^12^ conducted a study with 23 children comparing the combination of chloramphenicol and penicillin with intramuscular ceftriaxone. Both regimens resulted in a 100% cure rate, with ceftriaxone showing faster defervescence (Table 2).

Finally, the study by Pécoul et al.,^13^ conducted in West Africa with 528 patients, compared long-acting chloramphenicol (100 mg/kg in two intramuscular injections) with intravenous ampicillin (200 mg/kg/day). The cure rate was 72% in the chloramphenicol group and 75% in the ampicillin group. Chloramphenicol stood out for its practicality of administration in resource-limited settings (Table 2).

Cefuroxime has been extensively evaluated as an effective alternative to ceftriaxone, especially in settings where bacterial resistance or unavailability of ceftriaxone makes standard treatment difficult. These studies show that cefuroxime can achieve cure rates comparable to ceftriaxone, offering a viable alternative for the treatment of bacterial meningitis in children.

The multicenter study by Marks et al.^14^ included 107 children over 3 months of age, comparing cefuroxime (225 mg/kg/day) with the combination of ampicillin (300-400 mg/kg/day) and chloramphenicol (75-100 mg/kg/day). Clinical cure was achieved in 94% of patients in the cefuroxime group and in 93% in the ampicillin + chloramphenicol group. Both treatments demonstrated high efficacy in sterilizing the CSF, with minimal adverse effects and no significant clinical impact (Table 2).

In another study, Schaad et al.^15^ evaluated 171 children aged 3 months to 12 years, comparing ceftriaxone (100 mg/kg/day) with cefuroxime (200 mg/kg/day). Both treatments were highly effective, with cure rates of 94% and 92%, respectively. Although both demonstrated similar efficacy, ceftriaxone stood out for its convenience in a single daily dose, facilitating management in settings with limited resources. These studies indicate that, despite the small differences in convenience, cefuroxime represents a solid alternative, especially in situations where ceftriaxone is not available (Table 2).

Meropenem has been studied as a therapeutic option in contexts of high antimicrobial resistance, where standard treatments may not be effective. This antibiotic demonstrated great efficacy in bacterial eradication in children with bacterial meningitis, however, it presented some adverse effects, especially in patients with preexisting neurological conditions.

In a clinical trial conducted by Klugman et al.^16^ involving 190 children, meropenem (40 mg/kg administered every 8 hours) was compared with cefotaxime (75–100 mg/kg every 8 hours). Both regimens achieved complete bacterial eradication, demonstrating the potency of meropenem in resistant infection settings. This antibiotic is especially useful in cases of high resistance, such as infections caused by multidrug-resistant *S. pneumoniae*, where the use of ceftriaxone alone may not be effective. However, meropenem was associated with a higher incidence of seizures, especially in children with preexisting neurological disorders, an important factor to consider when choosing treatment. These findings suggest that, despite its high antimicrobial potential, meropenem should be used with caution in neurosensitive patients, prioritizing it in cases where other therapeutic options are limited or ineffective (Table 2).

Cefepime and cefotaxime have been evaluated as viable alternatives to ceftriaxone, especially in cases of bacterial meningitis where standard treatment may not be adequate or where a specific therapeutic response is required. These antibiotics have been shown to be effective in specific situations, especially in cases of infection by *H. influenzae* type b.

The multicenter study by Peltola et al.,^17^ involving 220 children, compared the use of chloramphenicol, ampicillin, cefotaxime and ceftriaxone. Both ceftriaxone and cefotaxime were shown to be significantly more effective in rapidly sterilizing cerebrospinal fluid (CSF), particularly in cases of meningitis caused by *H. influenzae* type b. Although both drugs showed high efficacy, ceftriaxone offered the advantage of convenient administration in a single daily dose, which facilitates management in clinical settings with limited resources. However, a higher incidence of mild diarrhea associated with the use of ceftriaxone was observed, an effect that, although mild, should be monitored (Table 2).

In another study, Sáez-Llorens et al.^18^ compared the efficacy of cefepime (50 mg/kg every 8 hours) and cefotaxime (50 mg/kg every 6 hours) in 90 children. Both treatments achieved cure rates of over 90%, with no significant differences in clinical outcomes, including neurological complications. These results indicate that, in addition to cefotaxime, cefepime may also be a safe and effective alternative to ceftriaxone, with an adequate safety profile and high efficacy in the treatment of bacterial meningitis (Table 2). Recent studies suggest that the duration of treatment with ceftriaxone can be reduced without compromising its efficacy, which is especially advantageous in resource-limited settings or where adherence to treatment may be challenging. This adjustment in the duration of the regimen can simplify clinical management and reduce costs without compromising clinical outcomes.

In the study by Molyneux et al.,^19^ conducted with 348 newborns, the regimens of benzylpenicillin and gentamicin with ceftriaxone were compared in the treatment of serious bacterial infections. Both regimens showed similar efficacy, with mortality rates of 13.7% for the regimen with benzylpenicillin and gentamicin and 16.5% for the regimen with ceftriaxone, with few differences in neurological complications. These results demonstrate that ceftriaxone can be used effectively in a short-term regimen without compromising safety, especially in serious pediatric cases (Table 2).

In turn, the study by Vaswani et al.^20^ involved 96 children and compared two treatment regimens with ceftriaxone and vancomycin, with durations of 7 and 10 days. Both groups had similar treatment success rates, with the 7-day regimen proving as effective as the 10-day regimen. These findings support the possibility of using a shorter course of ceftriaxone as a safe and effective alternative, offering a practical and cost-effective option for the treatment of pediatric bacterial meningitis, especially where long-term access to treatment is limited (Table 2).

## Discussion

This systematic review provides a comprehensive analysis of the efficacy and safety of alternative antibiotics in the treatment of childhood bacterial meningitis, offering valuable contributions to clinical practice and future research. Ceftriaxone was the most widely evaluated antibiotic and demonstrated high clinical and bacteriological efficacy, as well as a favorable safety profile. However, comparing these findings with current clinical guidelines and practices reveals not only confirmations but also important issues that deserve discussion.

Ceftriaxone, one of the most widely used antibiotics in the treatment of bacterial meningitis, has shown high efficacy in several reviewed studies. Studies such as that by Del Rio et al.^7^ demonstrated that ceftriaxone sterilizes cerebrospinal fluid (CSF) more rapidly than the combination of ampicillin and chloramphenicol, corroborating its superior efficacy. These findings are consistent with current guidelines from entities such as the World Health Organization (WHO) and the Centers for Disease Control and Prevention (CDC), which recommend ceftriaxone as the first-line antibiotic in many cases of bacterial meningitis, due to its efficacy and convenience of once-daily administration.^21^^,^^22^

More recent studies, such as those by Hathout et al.,^23^ Schaad et al.^24^ and Zar et al.,^25^ also emphasize the convenience of ceftriaxone, especially in resource-limited settings. However, when compared with treatment guidelines in developed countries, it is observed that some protocols prefer cefotaxime in certain situations, due to their more stable safety profile. This point reinforces the importance of personalizing treatment based on local clinical and epidemiological considerations. In addition to ceftriaxone, antibiotics such as cefotaxime and cefuroxime have shown comparable efficacy in clinical cure and CSF sterilization. Studies by Marks et al.^14^ and Schaad et al.^15^ indicate that cefuroxime may be a practical alternative, especially in settings where bacterial resistance or the availability of ceftriaxone is a limitation. Current guidelines reflect these options, recommending cefotaxime as an appropriate alternative in situations where ceftriaxone is not available or when the patient has allergies to this medication. Indeed, the Infectious Diseases Society of America (IDSA) guidelines include cefotaxime as a first-line option alongside ceftriaxone, especially for meningitis caused by *H. influenzae* type b.^26^

A key point raised in this review was the emergence of resistant strains, particularly of *S. pneumoniae*, which raises concerns about the use of ceftriaxone alone in regions where antimicrobial resistance is high.^27^^,^^28^ This highlights the need for continued surveillance and adjustments to guidelines based on local data on bacterial resistance. Modern guidelines from institutions such as the CDC already recommend regional monitoring of antimicrobial resistance and the use of broad-spectrum antibiotics, such as meropenem, only in cases of proven resistance.^29^ Although most of the studies reviewed showed a superiority of ceftriaxone over combination regimens, the study by Girgis et al.^10^ reported a complete recovery rate of 83% with ceftriaxone, while the ampicillin + chloramphenicol regimen achieved a considerable recovery of 74%. This suggests that, although ceftriaxone is effective, the use of combination therapies may be a valid alternative in settings where ceftriaxone is not available.

It is important to highlight that recent surveillance data from Brazil (2023) reported resistance to third-generation cephalosporins in over 30% of *S. pneumoniae* isolates from pediatric meningitis cases. This finding has led the Brazilian Society of Pediatrics to recommend empirical therapy with vancomycin combined with ceftriaxone for suspected pneumococcal meningitis until susceptibility profiles are confirmed. Despite vancomycin not being among the antibiotics evaluated in the included studies, its role in current empirical therapy is critical and must be considered in regions with high resistance rates. Therefore, the conclusions of this review should be interpreted within the limitations of the historical scope of the studies analyzed.^30^

The use of meropenem, a broad-spectrum carbapenem, was analyzed in the study by Klugman et al.,^16^ which showed 100% bacteriological efficacy, although it was associated with a higher incidence of seizures in children with pre-existing neurological disorders. These findings are particularly important considering clinical guidelines, which recommend the restricted use of carbapenems such as meropenem only in cases of severe bacterial resistance or failure of other antibiotics, due to its neurotoxic potential.^31^ The IDSA suggests that meropenem should be considered only in cases of bacterial meningitis due to multidrug-resistant pathogens, where third-generation cephalosporins such as ceftriaxone or cefotaxime have failed.^26^

Compared to other antibiotics, such as cefotaxime, which demonstrated a more stable safety profile in the study by Klugman et al.,^16^ meropenem should be used with caution and should be reserved for cases in which resistance to other antibiotics has already been confirmed or in patients who do not respond adequately to conventional treatments.

One of the most relevant findings of this review was the efficacy of shorter ceftriaxone regimens. Vaswani et al.^20^ suggest that a 7-day course is as effective as a 10-day regimen, with no increase in relapse rates or complications. This is in line with emerging discussions in clinical guidelines about the use of shorter antibiotic regimens, especially when adherence to treatment may be challenging. However, WHO and other guidelines, such as those from the National Institute for Health and Care Excellence (NICE), still recommend longer courses (10–14 days), highlighting the need for further studies before significant changes are made to widely established protocols.^22^^,^^32^

It is important to consider that although shorter regimens can be effective, as noted by Brink et al.,^33^ they may contribute to the emergence of antimicrobial resistance if not rigorously monitored. Therefore, clinical practice should balance the benefits of shorter courses with the potential risk of resistance, especially in hospitals facing a high prevalence of multidrug-resistant strains.

Similarly, the study by Molyneux et al.^19^ reinforced the efficacy of shorter regimens by comparing benzylpenicillin and gentamicin with ceftriaxone. The study showed that both regimens were equally effective, with similar mortality rates between groups. This finding suggests that ceftriaxone may be a practical and effective option in resource-limited settings, especially in developing countries, where costs and ease of administration are critical factors.

In addition, reducing the duration of treatment may contribute to minimizing the development of antimicrobial resistance, as noted by Brink et al.,^33^ which highlights the importance of shorter courses of antibiotics in hospitals facing the emergence of multidrug-resistant pathogens. The study by Sáez-Llorens et al.^18^ reinforces the safety of shorter courses by comparing cefepime and cefotaxime, showing that both antibiotics, administered for shorter periods, presented favorable clinical results. However, the emergence of multidrug-resistant strains of *S. pneumoniae* and *N. meningitidis* raises the need for caution when reducing the duration of therapy, as incomplete regimens may contribute to the development of resistance.

The safety of the reviewed antibiotics has been widely discussed and is generally considered satisfactory for the treatment of childhood bacterial meningitis. Ceftriaxone, for example, has demonstrated a favorable safety profile in several studies, being associated with mild adverse effects such as diarrhea and skin rash, as observed by Schaad et al.^15^ and Peltola et al.^17^ Although the incidence of mild diarrhea with ceftriaxone was slightly higher compared with other antibiotics, this adverse effect did not have a significant clinical impact, which reinforces its suitability as a first-line antibiotic, especially in resource-limited settings where once-daily administration is an advantage. Cefuroxime, evaluated in the studies by Marks et al.^14^ and Schaad et al.,^15^ also demonstrated a positive safety profile, being well tolerated and associated with few adverse effects, which makes it a practical option in settings where ceftriaxone is not available or where its safety is a concern.

On the other hand, the use of meropenem presented a more specific safety concern. In the study by Klugman et al.,^16^ although meropenem demonstrated high efficacy in bacterial eradication, it was associated with a higher incidence of seizures, especially in children with preexisting neurological conditions. This finding highlights the need for a cautious approach when using meropenem, reserving it for situations in which antimicrobial resistance renders conventional treatments ineffective. The neurotoxic potential of meropenem, which has been recognized in other guidelines, such as those of the Infectious Diseases Society of America (IDSA), reinforces that its use should be limited to cases of multidrug-resistant pathogens, where treatment alternatives are scarce. In summary, the review suggests that, although many of the antibiotics evaluated have an acceptable safety profile, the choice of treatment should always take into account the individual clinical history and the presence of comorbidities, particularly in cases that require the use of antibiotics with a greater potential for adverse effects, such as meropenem.

Despite the robustness of the findings, this review has several limitations. The heterogeneity of the included studies — both in terms of design and population characteristics — may introduce bias and hinder the comparability of results. Current clinical guidelines recommend long-term follow-up for children with bacterial meningitis, emphasizing the need to monitor for potential neurological sequelae. However, most studies included in this review reported only short-term outcomes, such as bacterial clearance and symptom resolution, without systematically assessing long-term complications.

These long-term complications — particularly neurological impairments and sensorineural hearing loss — are clinically significant and may vary depending on the antibiotic used. For example, meropenem has been associated with an increased incidence of seizures in children with underlying neurological conditions, while aminoglycosides, such as gentamicin, are known to carry a risk of ototoxicity, especially in neonates. The absence of standardized post-treatment follow-up protocols in most trials limits the ability to fully evaluate the long-term impact of antibiotic regimens. Future studies should incorporate validated tools to assess neurodevelopmental and auditory outcomes to ensure not only microbiological cure but also the preservation of cognitive and functional health.^34^^,^^35^

Additionally, although this review identified 14 eligible studies encompassing more than 2,000 pediatric patients, a formal meta-analysis was not conducted due to substantial methodological heterogeneity. This variability included differences in dosing regimens, treatment durations, definitions of outcomes (e.g., “clinical cure”), and the temporal context, as the studies span nearly four decades (1983–2021). The authors believe such heterogeneity could compromise the statistical validity and clinical interpretability of pooled estimates. Future systematic reviews may consider stratified meta-analyses once more homogeneous and contemporary data are available.

Another important consideration is the geographic distribution of the included studies. Most trials were conducted in high-income countries — particularly in Europe and North America — during the 1980s and 1990s. Only a limited number of studies originated from low- and middle-income countries (LMICs), where the current burden of pediatric bacterial meningitis is highest.

This imbalance affects the generalizability of the findings, as antimicrobial resistance patterns, access to diagnostics, and treatment protocols differ significantly across regions. For instance, although ceftriaxone remains an effective option in many settings, increasing resistance to third-generation cephalosporins—particularly in countries such as Brazil and India—necessitates a cautious interpretation of historical data. Therefore, the application of the present findings should be guided by regional surveillance data and context-specific clinical guidelines.^36^

## Conclusion

In conclusion, ceftriaxone remains a safe and effective first-line treatment for pediatric bacterial meningitis, particularly in resource-limited settings. However, alternative antibiotics such as cefuroxime and meropenem demonstrated comparable efficacy in specific contexts, especially in regions with a high prevalence of antimicrobial resistance. The use of shorter treatment regimens appears promising but should be implemented with caution and supported by robust clinical monitoring to avoid the emergence of resistance.

Notably, the increasing resistance of *Streptococcus pneumoniae* to third-generation cephalosporins—particularly observed in Brazil—has prompted updates in national guidelines recommending empirical combination therapy with vancomycin and ceftriaxone for suspected pneumococcal meningitis. As this review is based on historical clinical trials, its findings must be interpreted in light of current resistance trends and regional treatment protocols. Further long-term, well-designed studies are essential to evaluate both the effectiveness and safety of shortened regimens and their impact on neurological and auditory outcomes, ultimately guiding future updates in clinical practice.

## Conflicts of interest

The authors declare no conflicts of interest.

## Appendix Supplementary materials

Image, application 1

## Footnotes

Supplementary material associated with this article can be found in the online version at doi:10.1016/j.jped.2025.05.002.
