# Supplementary material for: Alternative antibiotics for the treatment of bacterial meningitis in children: a systematic review of efficacy and safety
Source: J Pediatr (Rio J). 2025 Jun 12;101(6):101414. doi: 10.1016/j.jped.2025.05.002 (PMC12744663; doi:10.1016/j.jped.2025.05.002)
Supplement: Supplementary file 1 [file mmc1.docx]

# **Appendix A** Search Strategies Used in the Databases.

| **Database** | **Search Terms Used** | **Applied Filters** |
| --- | --- | --- |
| **PubMed** | ("bacterial meningitis"[MeSH Terms] AND "antibiotic therapy"[MeSH Terms] AND "children"[MeSH Terms]) | Language: English  Study Type: Clinical Trials |
| **Scopus** | TITLE-ABS-KEY("bacterial meningitis" AND "antibiotic therapy" AND "children") | Language: English  Document Type: Article |
| **Web of Science** | TS = ("bacterial meningitis" AND "children" AND ("ceftriaxone" OR "chloramphenicol" OR "cefuroxime" OR "meropenem")) | Document Type: Article |
| **Cochrane Library** | "bacterial meningitis" in Title, Abstract, or Keywords AND "children" | Study Type: Clinical Trials |
